# Only Low Effects of Water Filters on the Enteric Carriage of Gastrointestinal Pathogen DNA in Colombian Indigenous People

**DOI:** 10.3390/microorganisms10030658

**Published:** 2022-03-19

**Authors:** Simone Kann, Gustavo Concha, Maria Hartmann, Thomas Köller, Juliane Alker, Ulrich Schotte, Lothar Kreienbrock, Hagen Frickmann, Philipp Warnke

**Affiliations:** 1 Medical Mission Institute, 97074 Würzburg, Germany; 2 Organization Wiwa Yugumaiun Bunkauanarrua Tayrona (OWYBT), Department Health Advocacy, Valledupar 2000001, Colombia; gustavoconcha16@gmail.com; 3 Institute for Biometry, Epidemiology and Information Processing, Veterinary Medical University Hannover, 30559 Hannover, Germany; maria.hartmann@tiho-hannover.de (M.H.); lothar.kreienbrock@tiho-hannover.de (L.K.); 4 Institute for Medical Microbiology, Virology and Hygiene, University Medicine Rostock, 18057 Rostock, Germany; thomas.koeller@med.uni-rostock.de (T.K.); frickmann@bnitm.de (H.F.); 5 Department A-Veterinary Medicine, Central Institute of the Bundeswehr Medical Service Kiel, 24119 Kronshagen, Germany; juliane.alker@gmail.com (J.A.); ulrichschotte@bundeswehr.org (U.S.); 6 Department of Microbiology and Hospital Hygiene, Bundeswehr Hospital Hamburg, 20359 Hamburg, Germany

**Keywords:** water sanitation, tropics, enteric pathogens, gut, stool samples, real-time PCR, hygiene, training, intervention, Colombia

## Abstract

Water filtration is a common strategy of water sanitation in resource-poor tropical settings. Here, we assessed the intermediate term effect of this preventive procedure including specific filter-related as well as general hygiene training on the molecular detection of enteric pathogens in stool samples from Colombian Indigenous people. From a total of 89 individuals from an Indigenous tribe called Wiwa, stool samples were assessed by real-time PCR for enteropathogenic microorganisms prior to the implementation of water filtration-based infection prevention. Three years after the onset of the preventive strategy, a follow-up assessment was performed. A significantly beneficial effect of water filtration could be shown for *Ascaris* spp. only (*p* = 0.035) and a tendency (*p* = 0.059) for *Hymenolepis nana*. No hints for effects on the gastrointestinal shedding of *Giardia duodenalis*, *Entamoeba histolytica*, *Cryptosporidium* spp., *Campylobacter* spp., *Shigella* spp./enteroinvasive *Escherichia coli*, *Necator americanus*, *Strongyloides stercoralis*, *Trichuris trichiura*, and *Taenia* spp. were seen. In conclusion, the study indicates that water filtration can only be an element of a multi-modal hygiene concept to reduce enteric pathogen carriage in inhabitants of resource-poor tropical settings in spite of tendencies of beneficial effects.

## 1. Introduction

As recently confirmed in a meta-analysis, access to clean water and sanitation facilitates the reduction of diarrhea-associated morbidity in resource limited settings [1]. One mode of ensuring increased water quality in resource-poor settings is the use of water filters. Indeed, water filtration can reduce diarrhea as repeatedly shown for various populations comprising not only children [2,3,4], but also HIV-(human immunodeficiency virus-) positive individuals and their relatives [5] or resource-poor households [6,7,8,9,10,11,12,13,14,15,16,17]. Gastroenteritis associated with water filter use was even demonstrated to slow down the disease progress of HIV infections [18,19,20] due to the associated lower immune-stimulation. As early as in the 1990s, the particular importance of water-borne infection prevention has been stressed by the US American Centers of Disease Control and Prevention (CDC) [21].

However, other studies on the preventive use of water filtration were less successful regarding the reduction of diarrheal disease [22], even in spite of reduced pathogen loads [23] and with varying effectiveness depending on seasonality [24]. In particular, interventions restricted to few sites such as increased water sanitation levels at school have been shown to be insufficient for health-related effects on the population level [25], while better effects can be seen if the implementation covers the homes of the children as well [26]. Sample size issues of the studies are other important features if convincing protective effects of water filtration cannot be shown [27]. Modelling approaches have also suggested that the effectiveness of filter-based water hygiene approaches will decline over time and can nearly vanish over a three years period [28]. Furthermore, the reliability of filter devices depends on their design and technical quality including the pore size of the filter in addition to other technical factors [29,30,31,32]. It must be pointed out that some studies showing protective effects of water filtration have worked with proxy parameters such as enteropathogen-induced serology only [33]. Furthermore, hygiene education and attitude towards the enforcement of hygiene precautions were also demonstrated to affect the protective effects associated with the implementation of water filtration strategies [34]. The integration of filter devices in a more comprehensive public health campaign has been suggested as advisable [35]. Altogether, compliance is a critical factor for the effectiveness of water hygiene procedures as suggested in a recent Cochrane review [36]. In a Bolivian study with only borderline significant effects of water filtration, limited adherence was identified as an interfering factor [37] and other assessments reported compliance limitations as well [38]. Not all water hygiene approaches in low-and-middle-income countries are equally effective, but water filtration is still among the most effective ones [39]. Filtration-based water hygiene is also very cost-efficient, increasing its interest for resource limited settings [40].

The first promising results of effective water filtration implementation were reported for tropical Colombia more than 15 years ago [41]. In the study provided here, implementation of water filtration was conducted in an Indigenous Colombian tribe called Wiwa, accompanied by specifically filter-related and general hygiene training, to improve water hygiene. Beneficial effects were assessed by the comparison of molecular detection rates of diarrhea-associated pathogens in their stool samples.

## 2. Materials and Methods

### 2.1. Study Design

The assessment was conducted as an interventional study based on the comparison of data from two cross-sectional analyses, which were performed within a time span of three years. In total, 220 water filters were distributed in four villages in resource-poor Colombian areas inhabited by the Wiwas. One filter was given to each head of a family, accompanied by a personal training of this person in water filter application and cleaning. In parallel, educational sessions on the topic of general and water hygiene were provided in all four villages. Additional water filters were provided to public institutions such as schools, kindergartens, health points, community kitchens, etc.

**Figure 1:**
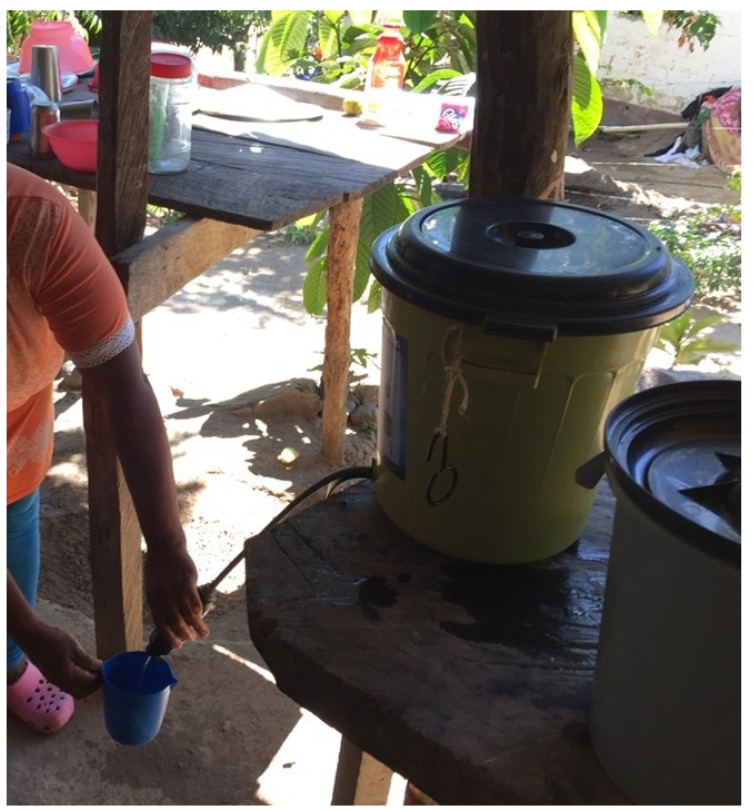
Installed water filter in a remote village inhabited by the Wiwas.

The applied water filters were purchased from the company Sawyer SAS (Medellín, Colombia). In detail, the filter type “Sawyer Point One TM Filters with the bucket adapter kit institutional (SP 180 ND)” was used. An installed filter is shown in Figure 1.

The filter consists of robust hollow fiber membranes, which tolerate filtering and backwashing (60 PSI forward and 20 PSI backward). All pores are uniform in size and no larger than 0.1 micron. According to the manufacturer, the filter can clean the water from 99.99% bacteria (evaluated for *Vibrio cholerae*, *Clostridium botulinum*, *Salmonella enterica* ssp. *enterica* ser. Typhi, *Escherichia coli* and others) as well as protozoan cysts (e.g., *Giardia duodenalis*, *Cryptosporidium* spp. and *Cyclospora cayetanensis*) and is able to filtrate 540 L per day. The filters were installed and applied exactly according to the manufacturer’s protocol (for details, see online resources at https://www.sawyer.com/products/international-bucket-system; https://www.youtube.com/watch?v=Ck7RZ6cKM7Y; https://www.sawyer.com/resources/sawyer-bucket-filtration-system-cleaning-instructions, all URLs last checked on 26 February 2022). The maintenance procedures that were demonstrated to the Wiwas comprised cleaning the filter every 2–3 days by turning it upside down and flushing it with freshly filtered water. In the case of very frequent use, daily cleaning was recommended. Applying this procedure, such filters can last for approximately 10 years as stated by the manufacturer. The Wiwas were taught to perform the cleaning procedure away from rooms such as the kitchen where food is handled in order to avoid contamination.

A total of 92 Colombian Indigenous people from Tezhumake (*n* = 40), Cherua (*n* = 36), Seminke (*n* = 12) and Ashintukwa (*n* = 4) provided stool samples before and three years after the implementation of the filter system. In the case of pathogen detections, pathogen-specific therapy was offered at both diagnostic assessment time points before and after the implementation of the filters but without diagnostic therapy control. Recorded epidemiological baseline information comprised age and sex. Adherence with the use of the filters was not systematically assessed but credibly reported by the Wiwas. The filtration procedure was technically evaluated after three years and the performance was still in line with requirements according to the manufacturer’s protocol in all households assessed.

### 2.2. Diagnostic Assessments

Stool samples were collected and stored at −20 °C in Colombia. After their shipment on dry ice with a specialized company (World Courier, Hildesheim, Germany) in line with international airfreight protocols, the stool samples were stored at −80°C prior to nucleic acid extraction, the same applied to the eluates after nucleic acid extraction. Due to changes in the diagnostic procedures during the three year duration of the study, the applied nucleic acid extraction procedures and real-time PCRs were different for the assessments before and after the implementation of the water filters. Diagnostic accuracy parameters of the used assays were, however, comparable as reported elsewhere [42,43]. The nucleic acids of the samples collected prior to the implementation of water filtration were extracted using the QiaAMP DNA stool mini kit (Qiagen, Hilden, Germany) as described by the manufacturer and assessed by in-house real-time PCR assays targeting entero-invasive bacteria as well as enteropathogenic protozoa and helminths exactly as described before [42,43,44,45]. The oligonucleotides applied for these in-house assays are provided in the Appendix A Table A1. The stool samples collected after the implementation of water filtration were subjected to nucleic acid extraction applying the automated Nimbus extractor (SeeGene, Seoul, Korea) followed by commercial real-time PCR with the Allplex GI-Bacteria(I), the Allplex GI-Parasite and the Allplex GI-Helminth(I) assays (SeeGene, Seoul, Korea). As a consequence of the use of different assays, PCR results were qualitatively assessed, while cycle threshold (Ct) values were not compared. Molecular diagnostic assessments were performed in the laboratories of the Institute for Medical Microbiology, Virology and Hygiene, University Medicine Rostock, in Rostock, Germany and of the Bernhard Nocht Institute for Tropical Medicine Hamburg in Hamburg, Germany. Both institutes are accredited according to DIN EN ISO 15189. Accordingly, standardized diagnostic quality can be considered as assured.

### 2.3. Statistical Assessments

For the statistical assessment, only individuals with PCR results at the sampling time points prior to and after the intervention were included. Due in part to insufficient sample volumes of individual samples for all assessments, the number of included samples per parameter varied over the different assessments (*n* = 89 vs. *n* = 78). Parameters were only included in the statistical assessment if they were part of the PCR panels applied before and after the intervention and if enough positive results were recorded to allow a sufficiently reliable statistical comparison. This applied for the protozoa *Giardia duodenalis*, *Entamoeba histolytica*, and *Cryptosporidium* spp., for the enteroinvasive bacteria *Campylobacter* spp., and *Shigella* spp./enteroinvasive *Escherichia coli* (EIEC), as well as for the helminths *Ascaris* spp., *Necator americanus*, *Strongyloides stercoralis*, *Trichuris trichiura*, *Taenia* spp., and *Hymenolepis nana*. Of note, *Shigella* spp./enteroinvasive *Escherichia coli* (EIEC)-PCR, which is based on the *ipaH* gene, does not allow further discrimination between these two pathogens. For the comparison of the pathogen DNA detections before and after the implementation of the water filters, the data were assessed calculating the simple kappa coefficient and applying McNemar’s test to identify statistical significance (accepted at *p* < 0.05).

### 2.4. Ethics

Ethical clearance for the study was provided by the Ethics Committee of the Tropical Health Foundation, Santa Marta (Acta No 032018, 02.04.2018) for the samples collected prior to the implementation of the water filters and by the Institutional Ethics Committee for Investigations of the University Area Andina, Valledupar (Acta No 2019-4, 29.10.19) for the assessment of the follow up samples. All participants had signed an informed consent form and received the results of their examinations, followed by a therapeutical offer if necessary. The study was in line with the declaration of Helsinki.

## 3. Results

### 3.1. Epidemiological Baseline Data from the Study Population

The Indigenous population lives in very retracted areas with sparse access to medical assistance. Long walking distances to the next health point (about 6 h) make them tolerate many complaints. In addition, during examinations by a physician in the course of this study, only very few mentioned diarrhea or abdominal pain as a problem, although various pathogens were found. Most of the stool samples showed a pathologic consistency, but were judged from the study participants as being normal.

Due to their territories being far away from the next hospital, only few hospital records are available. As diarrhea is seen as a “normal” condition by the Indigenous, only few of the Wiwas consulted a physician for this reason or even visited a hospital. As there is usually no doctor in place and the care is undertaken by sporadically appearing health brigades, whose members perform prevention care in the first line (vaccinations and tooth care), the Wiwas have little access to medical care and medication (e.g., antibiotics).

As the Wiwas are mainly agriculturists, they live from their earnings in the field. Their diet consists mainly of yuca, rice and cooked banana combined with eggs, meat and fish, if they can afford the latter. Among the consumed vegetables, beans and lenses are the main components.

Among the 92 study participants who provided stool samples before and three years after the implementation of the water filter-based prevention program, the male:female ratio was 48:44. The age distribution comprised 24 individuals lower than 6 years of age, 36 individuals in the age range between 6 and 18 years as well as 32 individuals older than 18 years.

### 3.2. Diagnostic Results

After the exclusion of three individuals who were lost to follow up, 89 individuals could be included in the assessments of the parameters *G. duodenalis*, *E. histolyica*, *Cryptosporidium* spp., *Campylobacter* spp., *Shigella* spp./EIEC, *Ascaris* spp., *Necator americanus*, and *Strongyloides stercoralis*. Due to sample volume limitations, only 78 individuals were included in the assessments of the additional parameters *T. trichiura*, *Taenia* spp. and *H. nana*. A detailed presentation of the results is provided in Table 1.

Altogether, little effect of the filter application was observed. For the majority of the analyzed parameters, at least slightly more lost infections than newly acquired infections were recorded. *Shigella* spp./EIEC and *Taenia* spp. were exceptions from this rule. A significant benefit of the use of the water filters, however, could be shown for *Ascaris* spp. only, with a weak significance of *p* = 0.035. For *H. nana*, at least a tendency for a beneficial effect (*p* = 0.059) was demonstrated, although significance was missed. All other recorded changes were most likely at random as suggested by McNemar’s test.

## 4. Discussion

The study did not show statistically significant beneficial effects of water filtration on infections with most of the assessed pathogens and even the single significance for an apparent improvement regarding *Ascaris* spp. infections would have been lost if correction for multiple testing with, e.g., the Bonferroni–Holm procedure [42], had been performed. This finding is in seeming contradiction to previous promising findings regarding the preventive effect of water filtration as reported in Colombian [41] and international studies [2,3,4,5,6,7,8,9,10,11,12,13,14,15,16,17]. In the previous Colombian assessment [41], the authors had reported on 60% less diarrhea due to filter-based prevention compared to a control population. However, the data are not directly comparable, as our study did not have a control group and pathogen DNA instead of symptoms was assessed. Furthermore, not all studies reported successful implementations [22,23,24]. In those studies from Kenya, the Democratic Republic of the Congo and Honduras, not even statistically significant reductions of clinical diarrhea could be shown. Notably, the studies indicating successful implementation usually used different endpoint parameters to the ones chosen for our assessment such as, e.g., the frequency of clinically apparent diarrhea or the microbiological quality of the consumed water [2,3,4,5,6,7,8,9,10,11,12,13,14,15,16,17]. The challenge of this study was considerably more complex, because real-time PCR from stool samples is a highly sensitive diagnostic approach, which is able to identify even traces of the pathogens’ nucleic acids [46]. Thereby, it is even difficult to discriminate infections from harmless colonization with enteropathogenic microorganisms, which has been reported to be a frequent phenomenon in tropical settings [46,47]. As clinical diarrhea was not assessed as part of the study protocol, it is unfeasible to define whether or not there has at least been a shift from clinical infection to harmless but yet PCR-based detectable colonization as a consequence of the implementation of water filtration. In addition, as diarrhea is considered as the “normal” stool consistency in these communities, which is due to various and persistent infections [48], medical history regarding gastrointestinal conditions is unlikely to be a good parameter for successful measurement.

In spite of this admitted limitation, the study clearly indicates that the sole implementation of water filters, including comprehensive hygiene training, does not relevantly reduce the overall abundance of DNA of enteric pathogens in stool samples in Indigenous people in remote Colombian settings. In spite of a tendency towards the right direction, as suggested by the mostly reduced numbers of pathogen DNA detections after the implementation of water filtration, it can only be a single component in a combined approach towards a general improvement in hygienic living conditions. As a result of the study, it can be claimed that the distribution of filters accompanied by teaching and training alone proved to be insufficient. At least, the implementation of filters was not associated with an increase of any assessed pathogens with the exception of *Taenia* spp. and *Shigella* spp./entero-invasive *Escherichia coli*. This latter finding, however, has to be interpreted with care, because a recent assessment [49] indicated reduced sensitivity of the *Shigella* spp./entero-invasive *E. coli*-specific in-house real-time PCR, which was applied prior to the distribution of the water filters. Therefore, it cannot be excluded that the initial prevalence was indeed higher but a higher detection limit of the screening PCR prevented an unbiased assessment regarding this parameter. Accordingly, we consider it justified to trust the general trend towards less pathogen DNA detections after the implementation of water filtration, in spite of the two observed contradicting parameters.

Of note, viruses were not assessed for the following reasons. First, the applied filters were not specifically designed for protection against viruses. Accordingly, obtained data would have been difficult to interpret. Second, due to the lack of local options of deep freezing at −80°C at the Colombian study site, optimum conditions for the preservation of viral RNA could not be ensured. Therefore, technical issues would also have interfered with the interpretation of the results of RNA virus-specific PCRs.

The study has several limitations. First and as previously shown [27], quantitative underpowering of studies on the effects of water hygiene is a likely reason why preventive effects cannot be demonstrated. Due to funding constraints, the number of included patients had to be restricted. However, strong effects would nevertheless have been visible, although overlooking of minor effects is theoretically possible. Second, different PCR assays were applied for the assessments before and after the implementation. Although previous assessments have suggested a generally good agreement of the applied assays as detailed elsewhere [42,43], minor bias effects regarding PCR signals at the diagnostic detection limit [49] cannot be excluded and previously observed differences between the recorded Ct values with the different assays [42,43] were the reason for abstaining from semi-quantitative assessments. Third, it may be speculated that better effects would have been achieved if more than one filter per family was distributed. Due to the limited numbers of filters, it is likely that water consumption from unsafe water sources during longer trips may have occurred as well, next to other potential sources of transmission such as contaminated food or soil, smear infections or lacking sanitary infrastructure. However, comparably high prices of the filters of about EUR 42 per device prevented a broader availability. Fourth, the lack of clinical data in the study protocol did not allow conclusions on potential clinical effects of the implementation of water filtration such as a shift from clinically apparent infections towards harmless colonization. As previously reported [48], however, complaints on gastrointestinal symptoms are socio-culturally discouraged among the Wiwas and therefore, respective medical history would most likely have been considerably biased. Fifth, the low number of included individuals did not allow stratification for potentially relevant factors such as age of the study participants. Sixth, due to the time period of three years, interfering environmental effects cannot be excluded. So, it can only be speculated if, e.g., seasonally altered environmental moisture or educative effects of hygiene training with the aim of avoiding top dressing in agriculture might have contributed to the observed beneficial effect on the enteric carriage of *Ascaris lumbricoides*. Seventh, without a matched control group without access to water filtration, definite conclusions are challenging, because water filter use might hypothetically have prevented even higher prevalence rates. In our study, only a historic control group was available.

## 5. Conclusions

As shown with stool samples of Colombian Indigenous people, water filtration, even if accompanied by hygiene training, is insufficient to drastically reduce the molecular detection rate of enteropathogenic microorganisms in spite of minor promising tendencies. Thus, water filtration and accompanying education can only be components in a more comprehensive multi-modal concept to improve the hygienic living conditions of these people.

## Figures and Tables

**Table 1:** Real-time PCR results before and after the water filtration intervention for diarrheagenic bacteria (a), enteric protozoa (b) and enteric helminths (c). A preponderance of the loss of detections over newly added detections is indicated in green, a situation without preponderance in one or the other direction in yellow and a preponderance of newly added detections over lost detections in red.

Real-Time PCR Parameter (Included Sample Count)	2018 and 2020 Concordantly Negatives	2018 and 2020 Concordantly Positives	2020 Newly Negatives (Which Were Still Positive in 2018)	2020 Newly Positives	Difference of Newly Negatives Minus Newly-Positives	Significance Level as Indicated by McNemar’s Test	Simple Kappa Coefficient (95% Confidence Interval)
(a) diarrheagenic bacteria							
*Campylobacter* spp. (*n* = 89)	44	14	16	15	1	*p* = 0858	0.214 (0.003, 0.425)
*Shigella* spp./EIEC (*n* = 89)	79	0	3	7	−4	*p* = 0.206	−0.050 (−0.091, −0.008)
(b) enteric protozoa							
*Entamoeba histolytica* (*n* = 89)	86	1	2	0	2	*p* = 0.157	0.491 (−0.109, 1.000)
*Giardia duodenalis* (*n* = 89)	15	41	19	14	5	*p* = 0.384	0.192 (−0.016, 0.400)
*Cryptosporidium* spp. (*n* = 89)	87	0	1	1	0	*p* = 1.000	−0.011 (−0.227, 0.004)
(c) enteric helminths							
*Ascaris* spp. (*n* = 89)	78	0	9	2	7	*p* = 0.035	−0.038 (−0.083, 0.006)
*Hymenolepis nana* (*n* = 78)	59	1	13	5	8	*p* = 0.059	−0.009 (−0.202, 0.185)
*Necator americanus* (*n* = 89)	80	1	6	2	4	*p* = 0.157	0.160 (−0.176, 0.497)
*Strongyloides stercoralis* (*n* = 89)	85	3	1	0	2	*p* = 0.317	−0.017 (−0.043, 0.009)
*Taenia* spp. (*n* = 78)	74	0	1	3	−2	*p* = 0.317	−0.020 (−0.049, 0.010)
*Trichuris trichiura* (*n* = 78)	73	0	4	1	3	*p* = 0.180	−0.021 (−0.054, 0.013)

## Data Availability

All relevant data are presented. Raw data can be made available upon reasonable request.

## Appendix A

**Table A1:** Genetic targets and oligonucleotide sequences of the applied in-house real-time PCRs (in alphabetic order) next to references detailing on the protocols. Adapted from [50].

Target Pathogen	Target Gene	Forward Primer Sequence	Reverse Primer Sequence	Probe Sequence	Reference, Providing More Details
*Ancylostoma* spp.	ITS2	GAATGACAGCAAACTCGTTGTTG	ATACTAGCCACTGCCGAAACGT	ATCGTTTACCGACTTTAG	[42]
*Ascaris lumbricoides*	ITS1	GTAATAGCAGTCGGCGGTTTCTT	GCCCAACATGCCACCTATTC	TTGGCGGACAATTGCATGCGAT	[42]
*Campylobacter jejuni*	*gyrA*	CTATAACAACTGCACCTACTAAT	AAGTGTAAGCACACAAGGTA	CTTAATAGCCGTCACCCCAC	[44]
*Cryptosporidium parvum*	138-bp fragment inside the *C. parvum*-specific 452-bp fragment	CGCTTCTCTAGCCTTTCATGA	CTTCACGTGTGTTTGCCAAT	CCAATCACAGAATCATCAGAATCGACTGGTATC	[42]
*Entamoeba histolytica*	SSU rRNA gene	ATTGTCGTGGCATCCTAACTCA	GCGGACGGCTCATTATAACA	TCATTGAATGAATTGGCCATTT	[42]
*Enterobius vermicularis*	ITS1	CGGTGTAATTTTGTTGGTGTCTATG	TGGCAGCATTGCAAACTAATG	TGTGCCAGTCAACGCCTAAACCGTC	[42]
*Giardia intestinalis*	SSU rRNA gene	GACGGCTCAGGACAACGGTT	TTGCCAGCGGTGTCCG	CCCGCGGCGGTCCCTGCTAG	[42]
*Hymenolepis nana*	ITS1	CATTGTGTACCAAATTGATGATGAGTA	CAACTGACAGCATGTTTCGATATG	CGTGTGCGCCTCTGGCTTACCG	[42]
*Necator americanus*	ITS2	CTGTTTGTCGAACGGTACTTGC	ATAACAGCGTGCACATGTTGC	CTGTACTACGCATTGTATAC	[42]
*Salmonella* spp.	*ttrC*	ATTGTTGATTCAGGTACAAAC	AATTAGCCATGTTGTAATCTC	CAAGTTCAACGCGCAATTTA	[44]
*Schistosoma* spp.	ITS2	GGTCTAGATGACTTGATYGAGATGCT	TCCCGAGCGYGTATAATGTCATTA	TGGGTTGTGCTCGAGTCGTGGC	[42]
*Shigella* spp./*Escherichia coli*, enteroinvasive (EIEC)	*ipaH*	CAGAAGAGCAGAAGTATGAG	CAGTACCTCGTCAGTCAG	ACAGGTGATGCGTGAGACTG	[44]
*Strongyloides stercoralis*	18S rRNA gene	GAATTCCAAGTAAACGTAAGTCATTAGC	TGCCTCTGGATATTGCTCAGTTC	ACACACCGGCCGTCGCTGC	[42]
*Taenia saginata*	ITS1	GCGTCGTCTTTGCGTTACAC	TGACACAACCGCGCTCTG	CCACAGCACCAGCGACAGCAGCAA	[42]
*Taenia solium*	ITS1	ATGGATCAATCTGGGTGGAGTT	ATCGCAGGGTAAGAAAAGAAGGT	TGGTACTGCTGTGGCGGCGG	[42]
*Trichuris trichiura*	18S rRNA gene	TTGAAACGACTTGCTCATCAACTT	CTGATTCTCCGTTAACCGTTGTC	CGATGGTACGCTACGTGCTTACCATGG	[42]
*Yersinia* spp.	*ail*	GCATTAACGAATATGTTAGC	ATCGAGTTTGGAGTATTCAT	CCGCTTCCAAATTTTGTCAT	[44]

bp = base pairs, ITS = internal transcribed spacer, rRNA = ribosomal ribonucleic acid, SSU = small subunit.
